# Microstructural, Magnetic, and Optical Properties of Pr-Doped Perovskite Manganite La_0.67_Ca_0.33_MnO_3_ Nanoparticles Synthesized via Sol-Gel Process

**DOI:** 10.1186/s11671-018-2553-y

**Published:** 2018-05-04

**Authors:** Weiren Xia, Heng Wu, Piaojie Xue, Xinhua Zhu

**Affiliations:** National Laboratory of Solid State Microstructures, School of Physics, Nanjing University, Nanjing, 210093 China

**Keywords:** (La_1 − x_Pr_x_)_0.67_Ca_0.33_MnO_3_ (LPCMO) nanoparticles, Perovskite manganites, Microstructural characterization, Spectral analyses, Magnetic properties, Optical bandgaps, Sol-gel process

## Abstract

We report on microstructural, magnetic, and optical properties of Pr-doped perovskite manganite (La_1 − x_Pr_x_)_0.67_Ca_0.33_MnO_3_ (LPCMO, *x* = 0.0–0.5) nanoparticles synthesized via sol-gel process. Structural characterizations (X-ray and electron diffraction patterns, (high resolution) TEM images) provide information regarding the phase formation and the single-crystalline nature of the LPCMO systems. X-ray and electron diffraction patterns reveal that all the LPCMO samples crystallize in perovskite crystallography with an orthorhombic structure (*Pnma* space group), where the MnO_6_ octahedron is elongated along the *b* axis due to the Jahn-Teller effect. That is confirmed by Raman spectra. Crystallite sizes and grain sizes were calculated from XRD and TEM respectively, and the lattice fringes resolved in the high-resolution TEM images of individual LPCMO nanoparticle confirmed its single-crystalline nature. FTIR spectra identify the characteristic Mn–O bond stretching vibration mode near 600 cm^− 1^, which shifts towards high wavenumbers with increasing post-annealing temperature or Pr-doping concentration, resulting in further distortion of the MnO_6_ octahedron. XPS revealed dual oxidation states of Mn^3+^ and Mn^4+^ in the LPCMO nanoparticles. UV-vis absorption spectra confirm the semiconducting nature of the LPCMO nanoparticles with optical bandgaps of 2.55–2.71 eV. Magnetic measurements as a function of temperature and magnetic field at field cooling and zero-field cooling modes, provided a Curie temperature around 230 K, saturation magnetization of about 81 emu/g, and coercive field of 390 Oe at 10 K. Such magnetic properties and the semiconducting nature of the LPCMO nanoparticles will make them as suitable candidate for magnetic semiconductor spintronics.

## Background

Perovskite manganites R_1 − x_A_x_MnO_3_ (R = La, Pr, and other rare earth elements, A = Ca, Sr, Ba, and other alkaline-earth elements) have received considerable interests during the past decade due to their colossal magnetoresistance (CMR) and potential applications in magnetic storage devices, magnetic sensors, and so on [1–3]. These materials exhibit interesting physical properties of concurrent ferromagnetism and metallic conductivity in the intermediate composition [2], which are ascribed to the complex interactions of the charge, orbital, spin, and lattice degrees of freedom [4–7]. La_1 − x_Ca_x_MnO_3_ (LCMO) manganite, as a prototypical system of perovskite manganites, has been of great interest because of its magnetic behavior and rich phase diagram [8, 9]. In the past decade, various synthesized methods such as sol-gel process [10, 11], polymeric precursor route [12], mechanical milling method [13], molten salt method [14] have been used to synthesize perovskite LCMO nanoparticles, and the effect of particle size on the structural, transport, and optical properties are also investigated [15–18]. Simultaneously, Ca-doped PrMnO_3_ (Pr_1 − x_Ca_x_MnO_3_: PCMO) also have some unusual electrical, magnetic, and optical properties, which are dependent on the Ca-doped concentration [19, 20]. As one typical representative of the partially substituted compounds in the Pr-doping La_1 − x_Ca_x_MnO_3_ (LPCMO) system, smaller cation Pr^3+^ replacing the larger cation La^3+^ has led to more interesting phenomena such as magnetocaloric effect and transverse Kerr effect [21, 22]. And also the LPCMO system is one of the most convenient ones for studying the phase separation behavior [23]. For example, TEM Uehara et al. [24] observed sub-micrometer sized phase separation involving ferromagnetic and charge-ordered antiferromagnetic domains with a typical size of about 0.2 μm in La_0.625 − y_Pr_y_Ca_0.375_MnO_3_. Furthermore, in the nanostructured LPCMO narrow strips (spatial confined system), several new transport features such as giant resistance jumps [25–27], reentrant M-I transitions [28], negative differential resistances, and intrinsic tunneling magnetoresistance [29, 30] were observed, which were absent in the counterparts of thin films and bulks. Hwang et al. [31] performed detailed studies on the transport and magnetic properties of Pr-doped manganite La_0.7 − x_Pr_x_Ca_0.3_MnO_3_ (*x* = 0.0–0.7), and they found that the transition temperature (*T*_*C*_) from paramagnetism to ferromagnetism phase was decreased monotonically and the magnitude of the magnetoresistance was enhanced dramatically. Cao et al. [32] studied the magnetic properties of La_0.67 − x_Pr_x_Ca_0.33_MnO_3_ (*x* = 0–0.67) synthesized by a conventional solid-state reaction, and found that the compounds underwent a ferromagnetic transition (*T*_*C*_) when the Pr-doping concentration (*x*) was below 0.4. Recently, Kumar et al. [33] performed studies on the structural, transport, and optical properties of the (La_0.6_Pr_0.4_)_0.65_Ca_0.35_MnO_3_ nanoparticles post-annealed at different temperatures. The optical bandgaps of the (La_0.6_Pr_0.4_)_0.65_Ca_0.35_MnO_3_ nanoparticles were deduced from their UV-vis absorption spectra, which were found to be ∼ 3.5 eV.

Up to now, the magnetic and transport properties of perovskite manganites have been widely investigated, whereas their optical properties are rarely reported since these systems exhibit either insulator behavior (with larger bandgaps over 4 eV) or metallic behavior (no bandgap). In this work, we report on the microstructural, magnetic, and optical properties of Pr-doped La_0.67_Ca_0.33_MnO_3_ nanoparticles [(La_1 − x_Pr_x_)_0.67_Ca_0.33_MnO_3_: LPCMO with *x* = 0.0–0.5] synthesized via a sol-gel process. The effects of the Pr-doping concentration and the post-annealed temperature on the structural, transport, and optical properties of perovskite LCMO nanoparticles are investigated systematically.

## Methods/Experimental

In this experiment, Pr-doped manganite (La_0.6_Pr_0.4_)_0.67_Ca_0.33_MnO_3_ nanoparticles were first synthesized via sol-gel process and post-annealed at 700, 800, 900, and 1000 °C. And then, perovskite (La_1 − x_Pr_x_)_0.67_Ca_0.33_MnO_3_ nanoparticles with *x* = 0.0–0.5 were synthesized by the same method and post-annealed at 800 °C. The starting materials were analytical grade La_2_O_3_, Pr_6_O_11_, CaCO_3_, and Mn(NO_3_)_2_·4H_2_O. First, at room temperature, analytical grade La_2_O_3_ powders and CaCO_3_ powders were dissolved in nitric acid with stirring. Simultaneously, analytical grade Pr_6_O_11_ powders were also dissolved in nitric acid with stirring and heating. Then, Mn(NO_3_)_2_·4H_2_O were added to the mixed solution of the above two solutions to form a solution of metal nitrates. To obtain the desired precursor solution, the solution of analytical grade citric acid and ethylene glycol prepared before were added to the former solution. Citric acid, ethylene glycol, and metal nitrates were prepared with a molar ratio of 4: 3: 1. The citric acid solution was used as a chelating agent while ethylene glycol was used as a gelification agent. After stirring for 10 min, the homogeneous precursor solution was dried in oven at 200 °C for 12 h to form the xerogel. The swelled xerogel was ground into powders and then was post-annealed at the temperature as mentioned above for 5 h with a heating rate of 5 °C/min. After heat treatment, the samples were cooled naturally to room temperature.

Phase identification of the LPCMO samples was performed by X-ray powder diffraction (XRD) at room temperature. The XRD data were collected from a Rigaku D/Max-RA diffractometer with Cu Kα radiation. A typical scan rate was 0.01^o^/s, and the 2θ range was 15^o^–85^o^. The average crystallite size (*D*) of the LPCMO samples was evaluated by using the Debye-Scherrer’s equation: *D* = 0.9λ/(βcosθ), where λ is the wavelength of Cu Kα radiation (λ = 1.5406 Å), β is the full width at half maximum intensity (FWHM) of the strongest XRD peak, and θ is the corresponding diffraction angle. The morphology and microstructure of the LPCMO samples were examined by analytical TEM (Tecnai G2S-Twin, FEI), and their compositions were determined by X-ray energy dispersive spectroscopy (EDS) (EX-250 spectroscopy, HORIBA Corporation). The specimens for TEM observations were prepared by drying droplets of the LPCMO powders from ethanol dispersion onto a holey carbon grid. Fourier transform infrared spectroscopy (FTIR) was performed with a FTIR Spectrometer (NEXUS870, Thermo Nicolet Corporation, USA) in the range of 400–4000 cm^− 1^ with a resolution of 1 cm^− 1^. The samples were mixed with KBr, and the pellets were prepared from the mixture. Raman spectroscopy measurements were carried out by using a Raman Spectrometer (LabRAM HR Evol, HORIBA Scientific, Japan) with visible laser light (wavelength 514.5 nm) as the excitation source. The slits were adjusted so that the resolution was 1 cm^− 1^. Room temperature XPS measurements were performed by a XPS Spectrometer (PHI 5000 Versa Probe, UlVAC-PHI, Japan). A MgKα anode was operated at 250 W, providing the excitation. The XPS spectra obtained were referenced to the referenced C ls peak (binding energy 284.60 eV). The absorption optical spectra of the LPCMO nanoparticles were measured in the range of 100–1000 nm by UV-vis spectrophotometer (UV2550, SHIMADZU, Japan) by using BaSO_4_ as a reference. The field and temperature dependence of magnetizations of the LCMO nanoparticles were measured by a SQUID magnetometer (Quantum design, America). First, the temperature was dropped from 300 to 2 K. The ZFC mode measurement data were collected with the temperature increasing from 2 to 300 K. And then, adding 0.01 T external magnetic field, the FC mode measurement data were collected with the temperature decreasing from 300 to 2 K.

## Results and Discussion

### Phase Identification of the LPCMO Nanoparticles

**Fig. 1:**
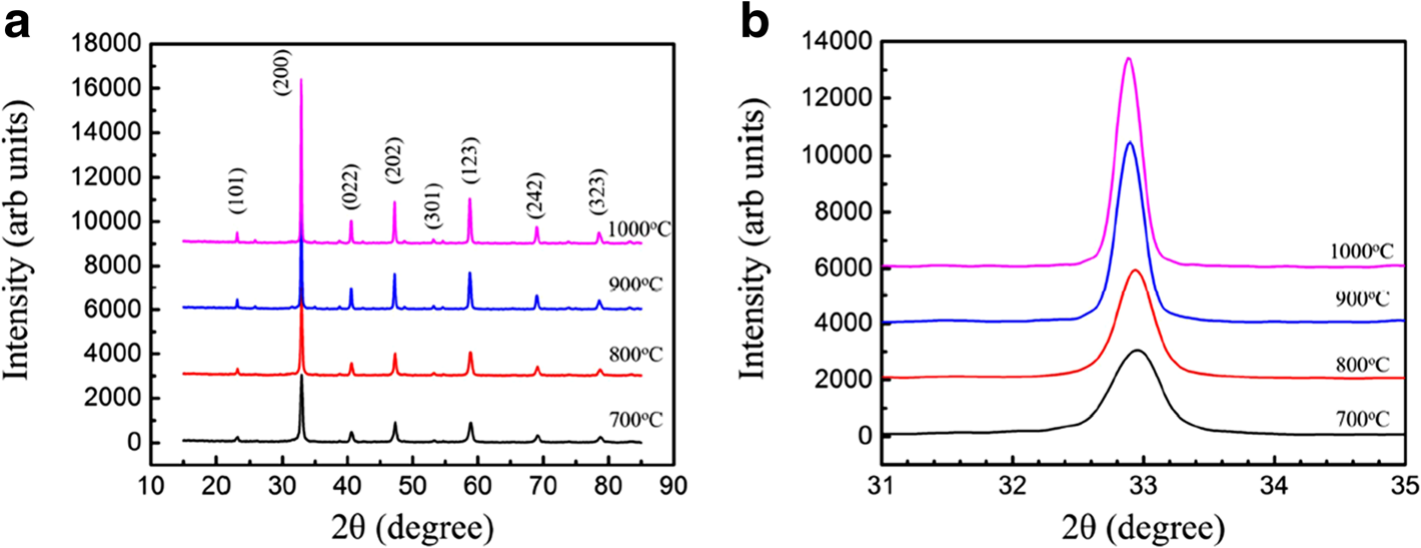
**a** X-ray diffraction patterns of the (La_0.6_Pr_0.4_)_0.67_Ca_0.33_MnO_3_ nanoparticles post-annealed at 700, 800, 900, and 1000 °C for 5 h. **b** Local (2θ = 31–35^o^) XRD patterns around the strongest diffraction peak (200)

The XRD patterns of the (La_0.6_Pr_0.4_)_0.67_Ca_0.33_MnO_3_ nanoparticles post-annealed at different temperatures (700–1000 °C) for 5 h are shown in Fig. 1a. It is found that all the diffraction peaks match well with the diffraction peaks of the La_0.67_Ca_0.33_MnO_3_ (JCPDS card no. 49-0416, *a* = 5.4515 Å, *b* = 7.7004 Å, *c* = 5.4671 Å, *α* = *β* = *γ* = 90^o^). That indicates all LPCMO samples have a single phase and there is no detectable secondary phase present. In fact, all the LPCMO samples crystallized in a single phase orthorhombic perovskite structure with space group *Pnma*. The lattice parameters and unit cell volumes of the LPCMO samples calculated from the XRD patterns are presented in Table 1. It was found that the lattice parameter *a* was generally increased with increasing the post-annealed temperature, which was confirmed by the left-shifting of the (200) diffraction peak, as shown in Fig. 1b. Meanwhile, the unit cell volumes of the LPCMO nanoparticles were also generally increased with increasing the post-annealed temperature. From the lattice parameters listed in Table 1, it is noticed that the lattice parameters (*a*, *b*, and *c*) satisfy a relationship of *a* ≈ *c* ≈ *b*/√2, indicating an orthorhombic distortion in perovskite crystallography [34]. The average crystallite sizes were determined by Scherrer’s equation, which were found to be 21, 32, 40, and 47 nm for the LPCMO nanoparticles post-annealed at 700, 800, 900, and 1000 °C, as listed in Table 1.

**Table 1:** The calculated lattice parameters (*a*, *b*, *c*, *α*, *β*, and *γ*), unit cell volumes, and average crystallite sizes of the (La_0.6_Pr_0.4_)_0.67_Ca_0.33_MnO_3_ nanoparticles post-annealed at (A) 700 °C, (B) 800 °C, (C) 900 °C, and (D) 1000 °C for 5 h. Lattice parameters within ± 0.0003(*a*, *b*, *c*) are calculated from the XRD data. Average crystallite sizes within ± 1 are calculated using the Scherrer’s equation

Sample number	Annealing temperature (°C)	Lattice parameters (Å)	Unit cell volume (Å^3^)	Average crystallite size (nm)
A	700	*a* = 5.4365, *b* = 7.6727, *c* = 5.4334 α = β = γ = 90^o^	226.64	21
B	800	*a* = 5.4411, *b* = 7.6940, *c* = 5.4213 α = β = γ = 90^o^	226.96	32
C	900	*a* = 5.4429, *b* = 7.6887, *c* = 5.4470 α = β = γ = 90^o^	227.95	40
D	1000	*a* = 5.4446, *b* = 7.6960, *c* = 5.4441 α = β = γ = 90^o^	228.12	47

**Fig. 2:**
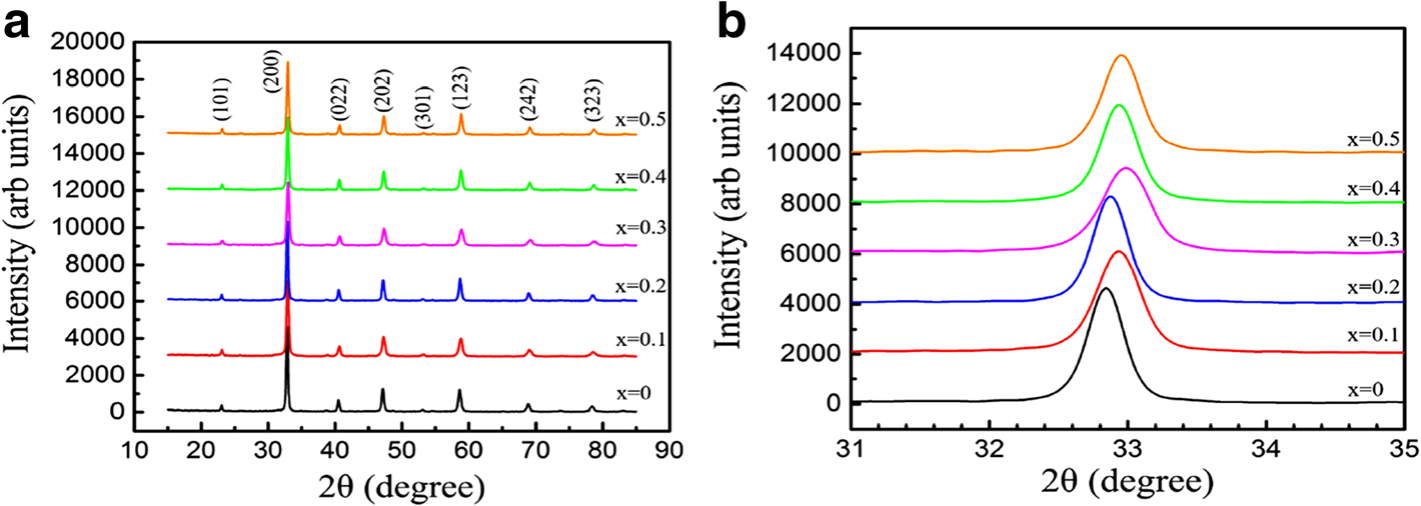
**a** X-ray diffraction patterns of the (La_1 − x_Pr_x_)_0.67_Ca_0.33_MnO_3_ (*x* = 0.0–0.5) nanoparticles post-annealed at 800 °C with different Pr-doping concentrations. **b** Local (2θ = 31–35^o^) XRD patterns around the strongest diffraction peak (200)

Figure 2a shows the XRD patterns of the (La_1 − x_Pr_x_)_0.67_Ca_0.33_MnO_3_ nanoparticles with different Pr-doping concentrations (*x* = 0.0–0.5), which were post-annealed at 800 °C for 5 h. Similarly, all the XRD data match well with the standard JCPDS card (no. 49-0416), indicating that all the samples crystallize in an orthorhombic perovskite structure. The lattice parameters and unit cell volumes calculated from the XRD patterns are listed in Table 2. The lattice parameters are also found to satisfy the relationship of *a* ≈ *c* ≈ *b*/√2, indicating a typical orthorhombic structural distortion in perovskite crystallography, where the MnO_6_ octahedron was elongated along the *b* axis due to the Jahn-Teller distortion in MnO_6_ octahedron [34]. It is also found that the lattice parameter *a* and the unit cell volumes of the samples have a slight decrease as increasing the Pr-doping concentrations. That is mainly attributed to the ionic radius of Pr^3+^ (99.0 pm) smaller than that of La^3+^ (103.2 pm). With increasing Pr-doping concentrations, the lattice parameter *a* was slightly decreased, leading to the right-shift of the (200) diffraction peak, as observed in Fig. 2b.

**Table 2:** The calculated lattice parameters (*a*, *b*, *c*, *α*, *β*, and *γ*), unit cell volumes, and average crystallite sizes of the (La_1 − x_Pr_x_)_0.67_Ca_0.33_MnO_3_ nanoparticles post-annealed at 800 °C with different Pr-doped concentrations (*x* = 0.0–0.5) Lattice parameters within ± 0.0003(*a*, *b*, *c*) are calculated from the XRD data. Average crystallite sizes within ± 1 are calculated using the Scherrer’s equation

Sample number	Pr-doped concentration (_X_)	Lattice parameters (Å)	Unit cell volume (Å^3^)	Average crystallite size (nm)
A′	0.0	*a* = 5.4624, *b* = 7.7114, *c* = 5.4539 *α = β = γ* = 90^o^	229.73	33
B′	0.1	*a* = 5.4302, *b* = 7.6815, *c* = 5.4505 *α = β = γ* = 90^o^	227.35	25
C′	0.2	*a* = 5.417, *b* = 7.6937, *c* = 5.4576 *α = β = γ* = 90^o^	228.91	31
D′	0.3	*a* = 5.4288, *b* = 7.6744, *c* = 5.4525 *α = β = γ* = 90^o^	227.17	21
E′	0.4	*a* = 5.4411, *b* = 7.6940, *c* = 5.4213 *α = β = γ* = 90^o^	226.96	32
F′	0.5	*a* = 5.4255, *b* = 7.6789, *c* = 5.4390 *α = β = γ* = 90^o^	226.60	27

### Microstructures of the LPCMO Nanoparticles

**Fig. 3:**
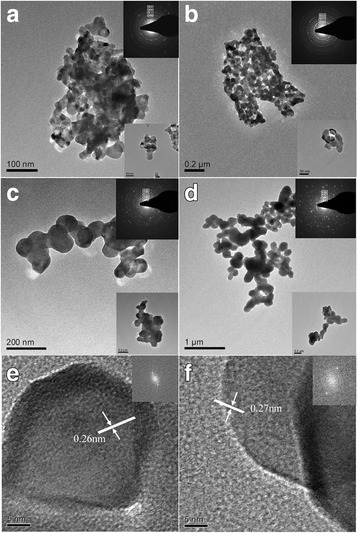
TEM images of the (La_0.6_Pr_0.4_)_0.67_Ca_0.33_MnO_3_ nanoparticles post-annealed at **a** 700 °C, **b** 800 °C, **c** 900 °C, and **d** 1000 °C for 5 h. Insets in (**a**–**d**) are the corresponding selected area electron diffractions taken from lots of (La_0.6_Pr_0.4_)_0.67_Ca_0.33_MnO_3_ nanoparticles, respectively. The indices are labeled based on the pseudo-cubic perovskite structure. **e**–**f** HRTEM images of the (La_0.6_Pr_0.4_)_0.67_Ca_0.33_MnO_3_ nanoparticle post-annealed at 700 and 900 °C for 5 h. Inset is the FFT pattern of the HRTEM images

TEM images of the (La_0.6_Pr_0.4_)_0.67_Ca_0.33_MnO_3_ nanoparticles post-annealed at different temperatures are shown in Fig. 3. As shown in Fig. 3a, the LPCMO nanoparticles are strongly agglomerated together due to the increased magnetic moment, which is ascribed to the suppression of antiferromagnetic ordering in the nanoparticles. Inset in Fig. 3a is the selected area electron diffraction (SAED) pattern taken from lots of the LPCMO nanoparticles, which exhibits polycrystalline diffraction rings consisting of discrete diffraction spots. The diameters (*D*_*i*_, *i* = 1–5) of the first five diffraction rings were measured, and the *D*_*i*_^2^*/D*_1_^2^ ratios were calculated. It is found that the *D*_*i*_^2^*/D*_1_^2^ ratios are equal to 1:2:3:4:6, which means that these diffraction rings are generated from an pseudo-cubic perovskite structure (in the pseudo-cubic setting), and the first five diffraction rings can be indexed as (101)_pc_, (200)_pc_, (211)_pc_, (220)_pc_, and (222)_pc_ (pc means the pseudo-cubic setting), respectively. With increasing the post-annealed temperatures, the LPCMO nanoparticles became less agglomerated, and their average crystallite sizes became increased (see Fig. 3c, d). The HRTEM images of the LPCMO nanoparticles post-annealed at 700 and 900 °C are shown in Fig. 3e, f, where the lattice fringes with inter-planar spacing of 0.26 or 0.27 nm are clearly resolved. These lattice fringes corresponds to the (200) lattice spacing of the orthorhombic perovskite (La_0.6_Pr_0.4_)_0.67_Ca_0.33_MnO_3_. Therefore, the single-crystalline nature of the LPCMO nanoparticles is proven by the lattice fringes resolved in the HRTEM images of individual LPCMO nanoparticles.

**Fig. 4:**
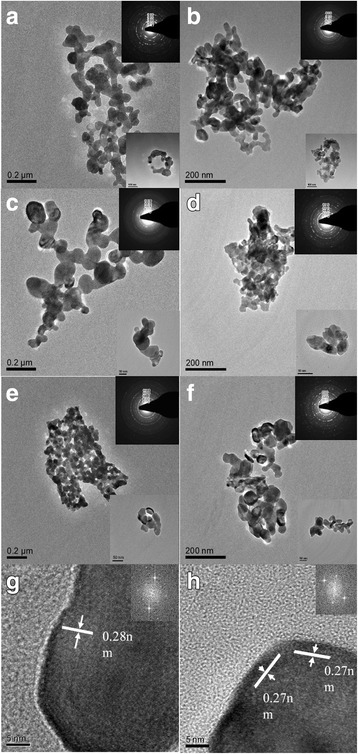
TEM images of the (La_1 − x_Pr_x_)_0.67_Ca_0.33_MnO_3_ nanoparticles post-annealed at 800 °C with different Pr-doping concentrations. **a** *x* = 0.0, **b** *x* = 0.1, **c** *x* = 0.2, **d** *x* = 0.3, **e** *x* = 0.4, and **f** *x* = 0.5. Insets in (**a**–**f**) are the corresponding SAED patterns of the (La_1 − x_Pr_x_)_0.67_Ca_0.33_MnO_3_ nanoparticles, respectively. The indices are labeled based on the pseudo-cubic perovskite structure. **g**–**h** HRTEM images of the (La_1 − x_Pr_x_)_0.67_Ca_0.33_MnO_3_ nanoparticles with Pr-doping concentration *x* = 0.1 and *x* = 0.3. Insets are the FFT patterns of the HRTEM images

Similarly, the TEM images of the (La_1 − x_Pr_x_)_0.67_Ca_0.33_MnO_3_ (*x* = 0.0–0.5) nanoparticles post-annealed at 800 °C for 5 h are shown in Fig. 4, which reveals that the LPCMO nanoparticles exhibit irregular granular shapes. The SAED patterns (see the insets) taken from many LCMO nanoparticles also exhibit the feature of diffraction patterns taken from polycrystalline nanopowders, where the polycrystalline diffraction rings are composed of the discrete diffraction spots. Based on their *D*_*i*_^2^*/D*_1_^2^ ratios of the polycrystalline diffraction rings, the first five diffraction rings can be indexed as (101)_pc_, (200)_pc_, (211)_pc_, (220)_pc_, and (222)_pc_, respectively. Figure 4g, h are the HRTEM images taken from single (La_1 − x_Pr_x_)_0.67_Ca_0.33_MnO_3_ nanoparticle with *x* = 0.1 and 0.3, respectively. The lattice fringes with inter-planar spacing of 0.27 or 0.28 nm are clearly resolved, which indicate the single-crystalline nature of the LPCMO nanoparticles. Electron dispersive X-ray spectra (EDS) of the LPCMO samples were also collected to determine their chemical compositions, and the quantitative EDS measurements from the LPCMO nanoparticles reveal that the cation atomic ratio of La: Pr: Ca: Mn were close to the desired stoichiometric proportions (not shown here).

### Spectra Analysis of the LPCMO Nanoparticles

**Fig. 5:**
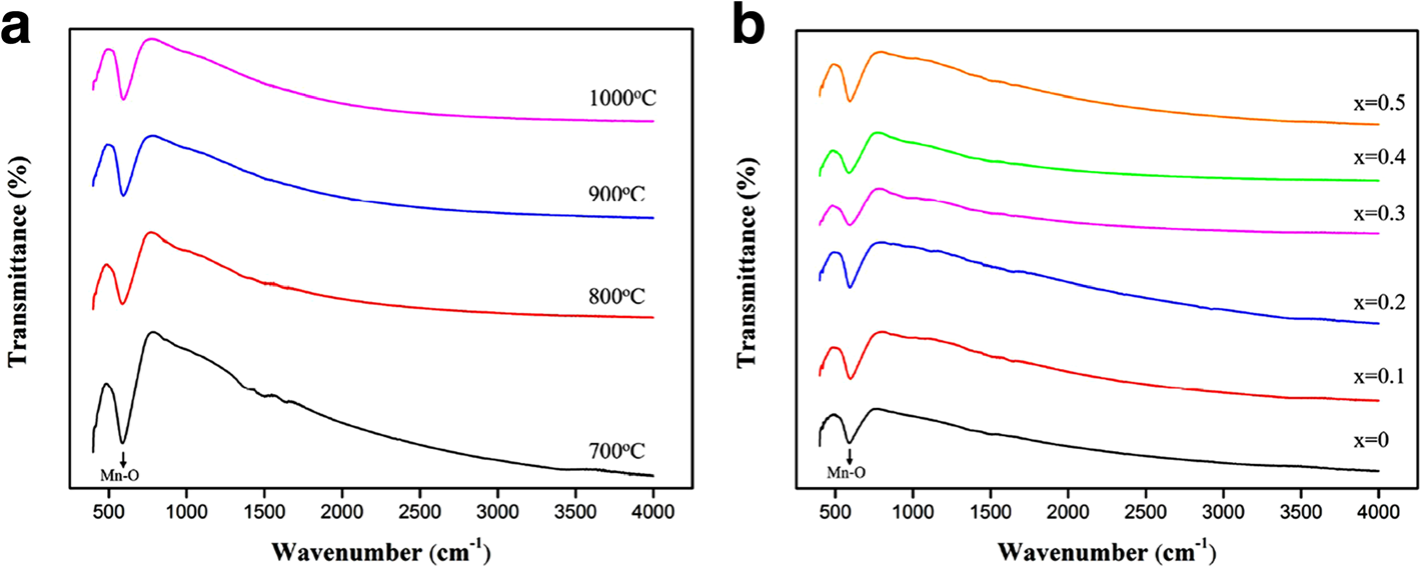
**a** Fourier transform infrared spectroscopy of the (La_0.6_Pr_0.4_)_0.67_Ca_0.33_MnO_3_ nanoparticles post-annealed at 700, 800, 900, and 1000 °C for 5 h. **b** Fourier transform infrared spectroscopy of the (La_1 − x_Pr_x_)_0.67_Ca_0.33_MnO_3_ nanoparticles post-annealed at 800 °C with different Pr-doping concentrations (*x* = 0.0–0.5)

Fourier transform infrared (FTIR) spectroscopy is used to investigate the lattice vibration in the present LPCMO system. Figure 5a shows the FTIR spectra of the (La_0.6_Pr_0.4_)_0.67_Ca_0.33_MnO_3_ nanoparticles post-annealed at different temperatures for 5 h, and Fig. 5b displays the FTIR spectra of the (La_1 − x_Pr_x_)_0.67_Ca_0.33_MnO_3_ (*x* = 0.0–0.5) nanoparticles post-annealed at 800 °C for 5 h. In Fig. 5, an obvious absorption peak in the frequency around 595 cm^− 1^ is observed in all the LPCMO nanoparticles, which can be attributed to the Mn–O–Mn bonds vibrating in the stretching mode [35]. This vibration mode is closely related to the change in the Mn–O–Mn bond length. With increasing the post-annealing temperature or the Pr-doping concentration, the stretching vibration mode frequency tends to move towards high wavenumbers (blue-shift) due to the reduction of the Mn–O bond length, indicating the further distortion of the MnO_6_ octahedron.

**Fig. 6:**
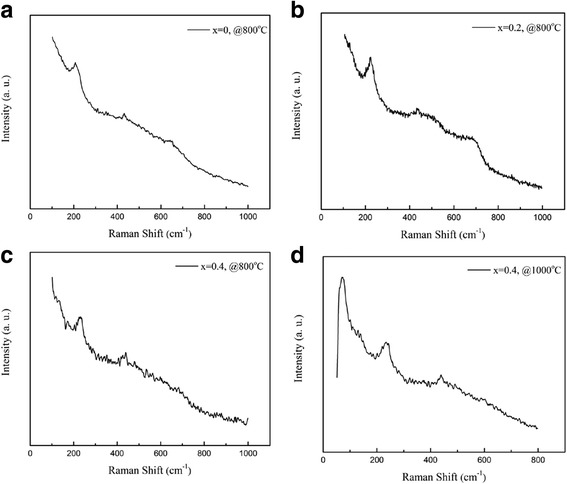
Raman spectra of the (La_1 − x_Pr_x_)_0.67_Ca_0.33_MnO_3_ nanoparticles with **a** *x* = 0.0 and **b** *x* = 0.2 and post-annealed at 800 °C, and with *x* = 0.4 and post-annealed at **c** 800 °C and **d** 1000 °C

Raman spectroscopy is also used to study the lattice distortions in the LPCMO nanoparticles. Figure 6 demonstrates the Raman spectra of the (La_1 − x_Pr_x_)_0.67_Ca_0.33_MnO_3_ nanoparticles post-annealed at 800 and 1000 °C. Three Raman peaks around 224, 425, and 680 cm^− 1^ are observed in Fig. 6a, b, respectively. The Raman peak around 224 cm^− 1^ can be assigned as A_g_(2), which is related to the tilting of MnO_6_ octahedron, whereas the Raman peak around 425 cm^− 1^ is related to the Jahn-Teller type modes of the MnO_6_ octahedron [33]. The Raman peak around 680 cm^− 1^ can be assigned as B_2g_(1), which is related to the symmetric stretching vibration of oxygen in MnO_6_ octahedron [33]. With increasing the Pr-doping concentration (*x*) up to *x* = 0.4, the Raman peak around 680 cm^− 1^ disappeared, as shown in Fig. 6c, d. That was ascribed to the increased orthorhombic distortion in the LPCMO nanoparticles with high Pr-doping concentrations, leading to the much weak symmetric stretching vibration of oxygen in MnO_6_ octahedron.

**Fig. 7:**
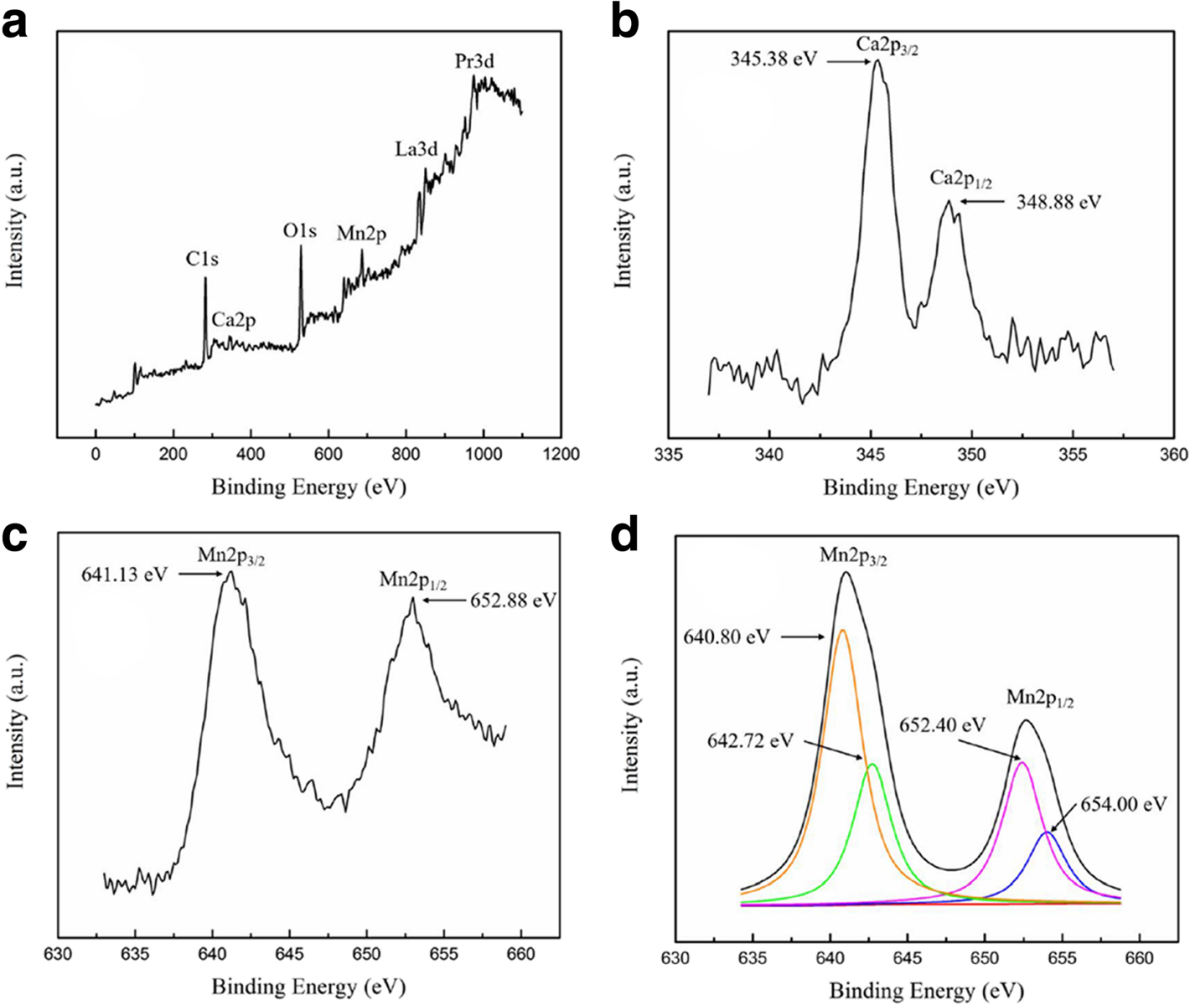
XPS spectra of the (La_0.6_Pr_0.4_)_0.67_Ca_0.33_MnO_3_ nanoparticles post-annealed at 800 °C for 5 h. **a** Survey scan spectrum. **b** Ca 2p. **c** Mn 2p XPS spectra. **d** Deconvoluted XPS peaks of the Mn 2p XPS spectra

In order to evaluate the ionic valence states in the LPCMO nanoparticles, especially Mn ions, XPS measurements were performed on the (La_0.6_Pr_0.4_)_0.67_Ca_0.33_MnO_3_ nanoparticles post-annealed at 800 °C for 5 h, and the results are shown in Fig. 7. As shown in Fig. 7a, a survey XPS scan of the (La_0.6_Pr_0.4_)_0.67_Ca_0.33_MnO_3_ nanoparticles reveals the La 3d, Pr 3d, Ca 2p, Mn 2p, and O 1s XPS peaks, indicating the existence of La, Pr, Ca, Mn, and O elements in the LPCMO nanoparticles. The observed C 1s XPS peak in this spectrum is probably due to the surface contamination in air. The narrow-scan XPS spectrum for Ca 2p from the LPCMO nanoparticle is shown in Fig. 7b, where two XPS peaks are located at 345.38 and 348.88 eV, which are assigned as Ca 2p_3/2_ and Ca 2p_1/2_, respectively due to the spin-orbit splitting of 3.5 eV. That indicates that Ca exists in + 2 oxidation state. Figure 7c shows the narrow-scan XPS spectrum of Mn 2p from the LPCMO nanoparticle, where two XPS peaks located at 641.13 and 652.88 eV are assigned as Mn 2p_3/2_ and Mn 2p_1/2_, respectively. These two XPS peaks are further analyzed by XPS-peak-differentiation-imitating method. The deconvoluted peaks of the Mn 2p_3/2_ and Mn 2p_1/2_ XPS peaks are shown in Fig. 7d. Clearly, the Mn 2p_3/2_ XPS peak is deconvoluted into two peaks at 640.80 and 642.72 eV, corresponding to the Mn^3+^ and Mn^4+^ ions, respectively. Similarly, the Mn 2p_3/2_ XPS peak is also deconvoluted into two peaks at 652.40 and 654.00 eV, corresponding to the Mn^3+^ and Mn^4+^, respectively. As a consequence, the deconvoluted Mn 2p XPS peaks reveal the existence of dual two oxidation states of the Mn^3+^ and Mn^4+^ ions. The Mn 2p_3/2_ and Mn 2p_1/2_ core levels are split into two peaks due to two valences of manganese upon Ca^2+^ doping, which forms the basis of the double exchange interaction. In addition, the content ratio of the Mn^3+^ to Mn^4+^ ions estimated from the deconvoluted XPS peak areas was approximately 2:1.

**Fig. 8:**
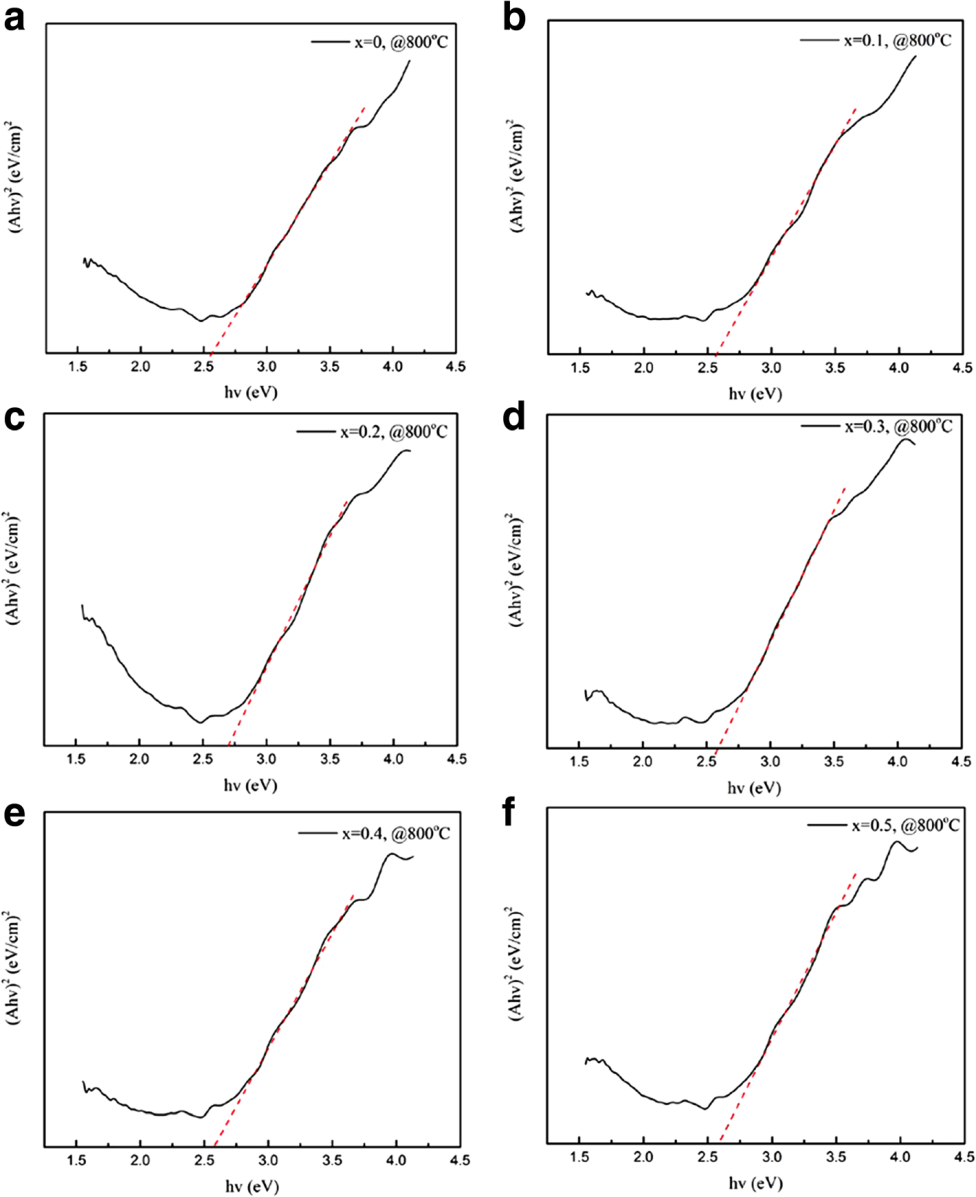
Variation of (*αhν*)^2^ versus photon energy (*hν*) for the (La_1 − x_Pr_x_)_0.67_Ca_0.33_MnO_3_ nanoparticles post-annealed at 800 °C with different Pr-doping concentrations. **a** *x* = 0.0, **b** *x* = 0.1, **c** *x* = 0.2, **d** *x* = 0.3, **e** *x* = 0.4, and **f** *x* = 0.5

Ultraviolet-visible (UV-vis) absorption spectra were measured to estimate the optical bandgaps of the (La_1 − x_Pr_x_)_0.67_Ca_0.33_MnO_3_ (*x* = 0.0–0.5) nanoparticles post-annealed at 800 °C for 5 h. The optical absorption edges can be analyzed as follows [36]:

$$ \upalpha hv\propto {\left( h\nu -{E}_g\right)}^n $$

where α is the absorption coefficient, depending upon the optical absorbance and thickness of the samples [36]. *n* can be equal to 1/2 (for direct transition process) or 2 (for indirect transition process). The plots of (α*hν*)^2^ versus the energy of photon (*hν*) for the LPCMO nanoparticles are shown in Fig. 8. A linear relationship between (α*hν*)^2^ and *hν* in a wide range is observed, suggesting a direct transition process taking place in the present system. The intercepts of these plots on the *hν* axis provide the optical bandgaps of the LPCMO nanoparticles, which are measured in the range of 2.55–2.71 eV (in the region of wide bandgap semiconductors), indicating the semiconducting nature of the LPCMO nanoparticles. The observed bandgaps of the LPCMO nanoparticles are smaller than that reported previously for the (La_0.6_Pr_0.4_)_0.65_Ca_0.35_MnO_3_ nanoparticles (~ 3.5 eV) by S. Kumar et al. [33]. The possible origins may be their different La/Ca ratios in the perovskite manganites and their different particle sizes.

### Magnetic Properties of the LPCMO Nanoparticles

**Fig. 9:**
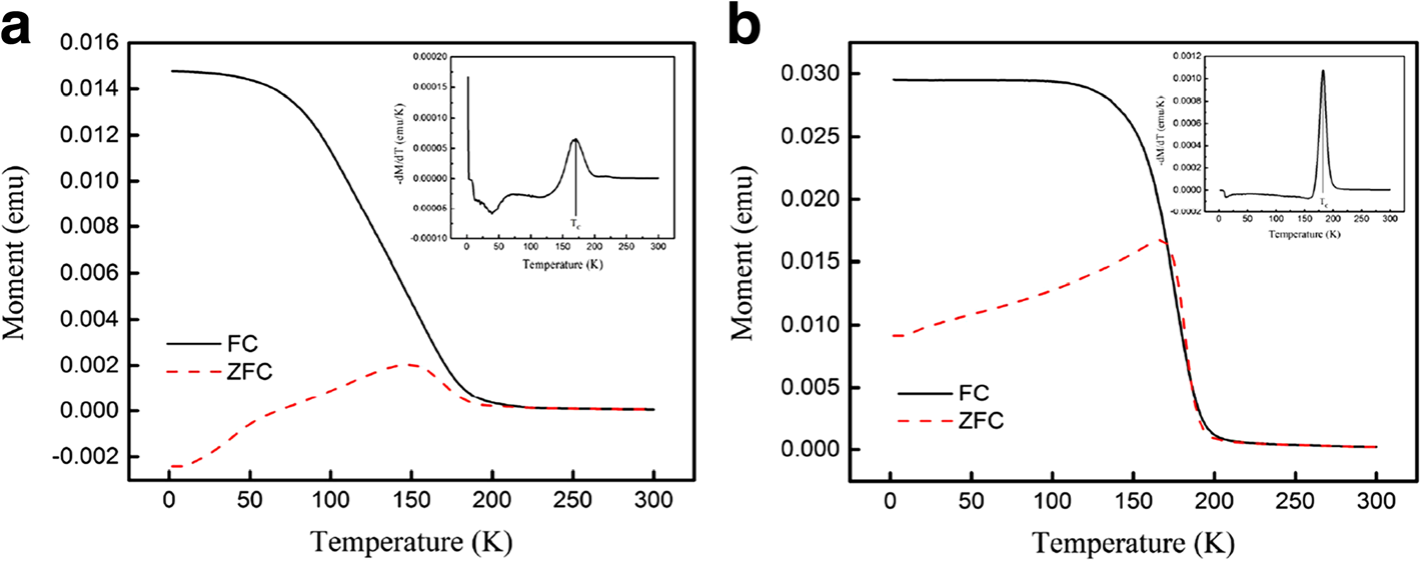
Temperature dependence of the magnetizations of the (La_0.6_Pr_0.4_)_0.67_Ca_0.33_MnO_3_ nanoparticles post-annealed at (**a**) 800oC and (**b**) 1000oC. Insets are the - dM/dT curves versus the temperature

**Fig. 10:**
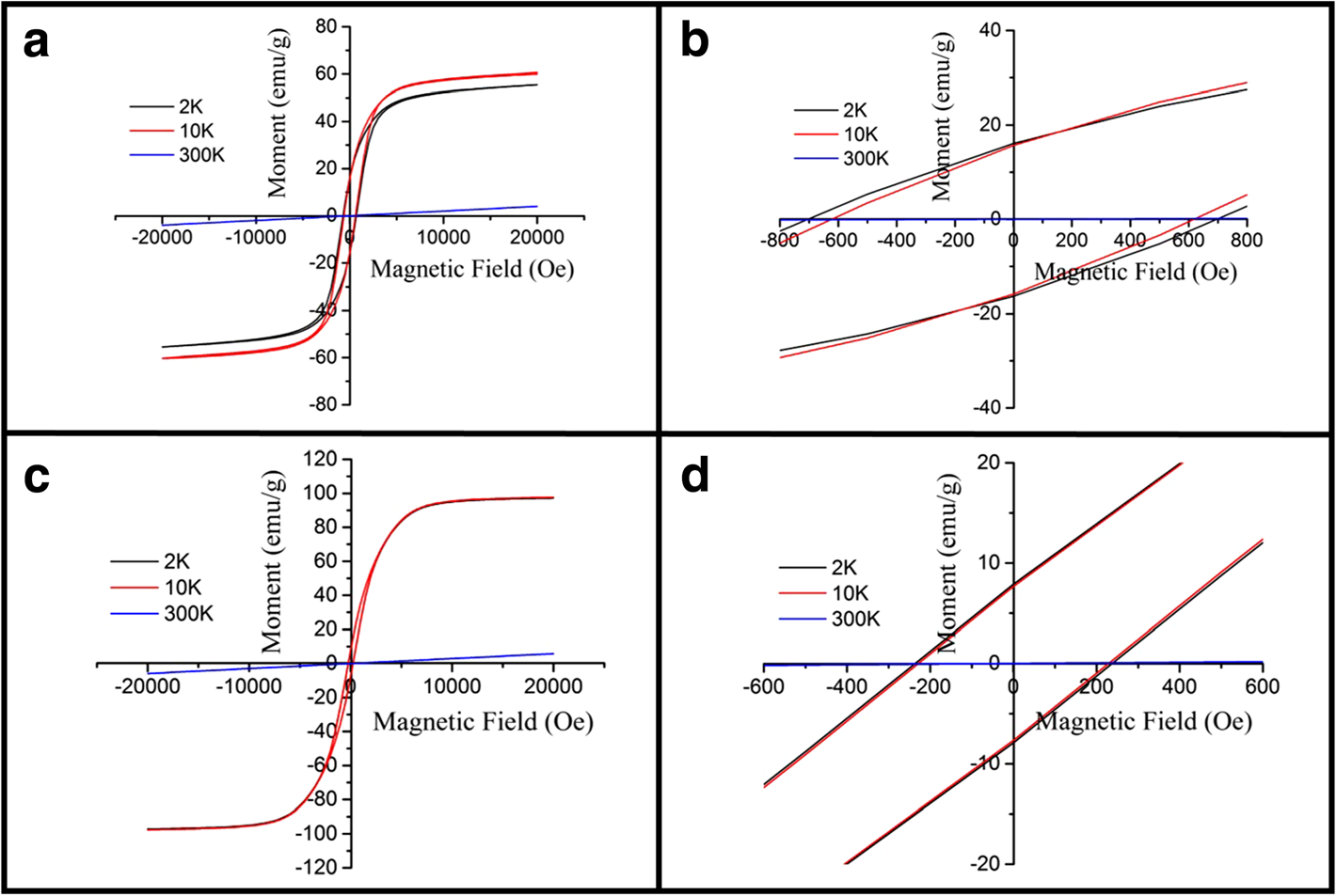
**a** and **c** M–H hysteresis loops of the (La_0.6_Pr_0.4_)_0.67_Ca_0.33_MnO_3_ nanoparticles post-annealed at 800 and 1000 °C, respectively. **b** and **d** are the corresponding local enlarged M–H hysteresis loops in (**a** and **c**), respectively

**Fig. 11:**
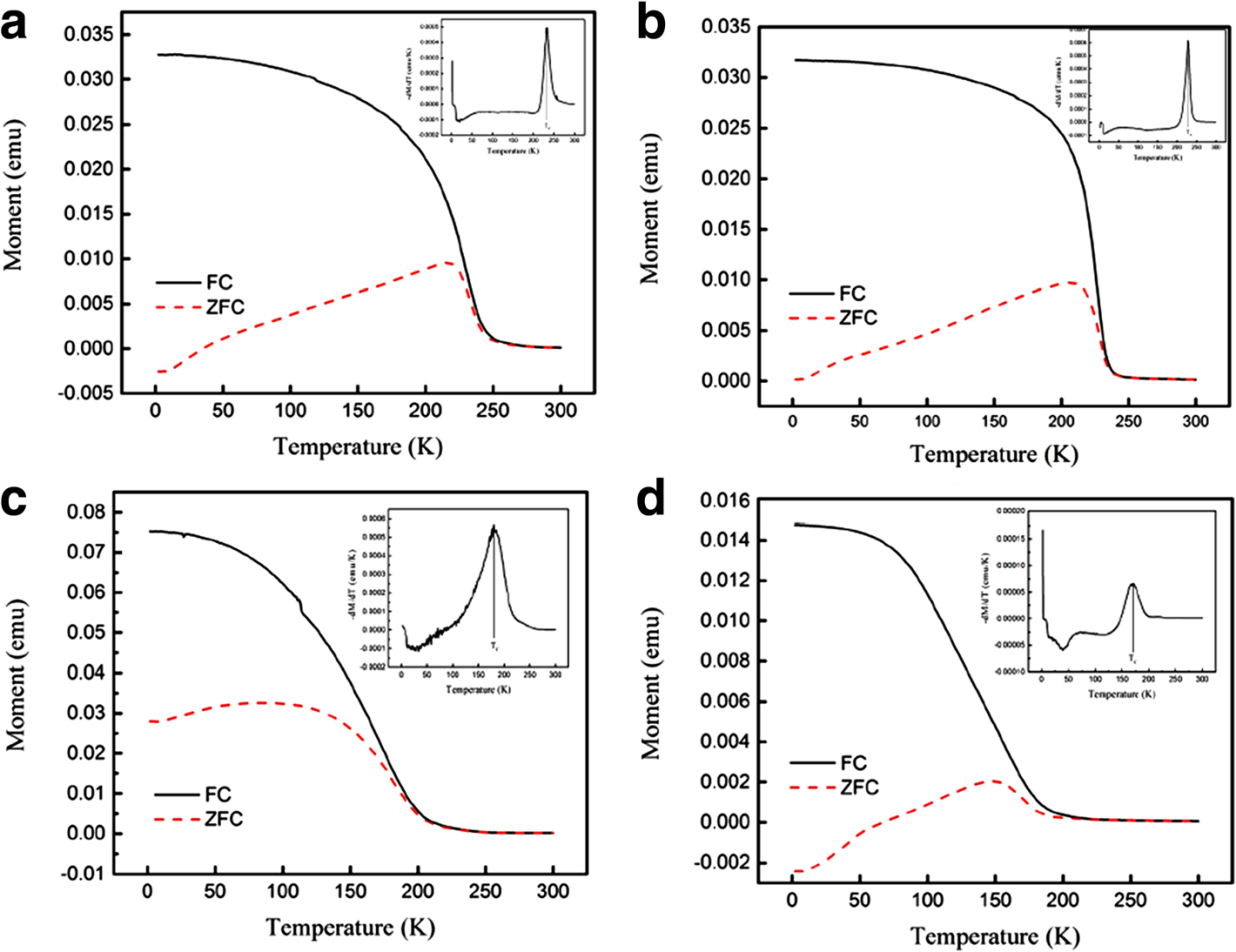
Temperature dependence of the magnetizations of the (La_1 − x_Pr_x_)_0.67_Ca_0.33_MnO_3_ nanoparticles post-annealed at 800 °C with different Pr-doping concentrations. **a** *x* = 0.1, **b** *x* = 0.2, **c** *x* = 0.3, and **d** *x* = 0.4

**Fig. 12:**
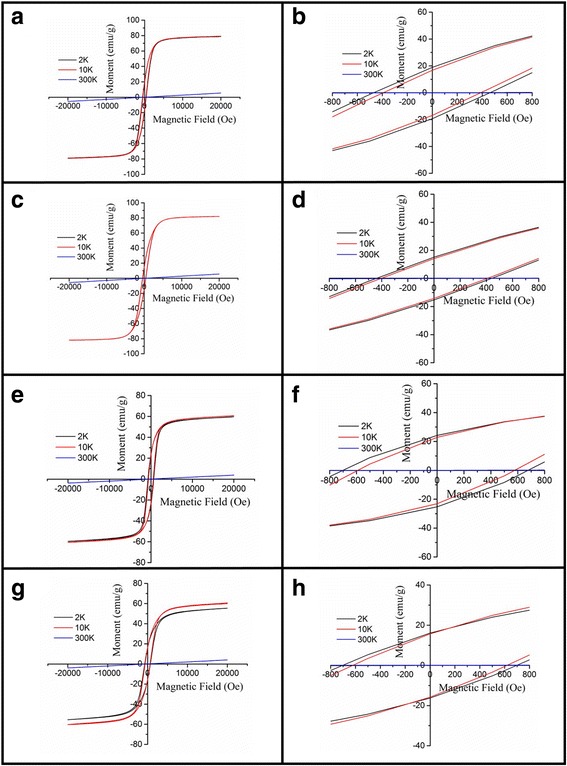
M–H hysteresis loops of the (La_1 − x_Pr_x_)_0.67_Ca_0.33_MnO_3_ nanoparticles post-annealed at 800 °C with different Pr-doping concentrations. **a** *x* = 0.1, **c** *x* = 0.2, **e** *x* = 0.3, and **g** *x* = 0.4. **b**, **d**, **f**, and **h** are the corresponding local enlarged M–H hysteresis loops in (**a**, **c**, **e**, and **g**), respectively

Temperature dependence of the magnetization *M*(*T*) of the (La_0.6_Pr_0.4_)_0.67_Ca_0.33_MnO_3_ samples post-annealed at 800 and 1000 °C is shown Fig. 9, which is measured at the zero-field cooling (ZFC) mode and field-cooling (FC) modes under an external magnetic field of 0.01 T. The M-T data demonstrated that all the LPCMO samples underwent a PM-FM transition upon cooling, and the Curie temperature *T*_*c*_ (defined as the one corresponding to the peak of − *dM/dT* in the *M* vs *T* curve) was determined to be 171 and 183 K for the samples post-annealed at 800 and 1000 °C, respectively. These values are close to that reported for the La_0.4_Pr_0.3_Ca_0.3_MnO_3_ sample (186 K) [37]. In addition, the *M*_FC_(*T*) of the (La_0.6_Pr_0.4_)_0.67_Ca_0.33_MnO_3_ samples exhibits almost constant value as further decreasing the temperature, and a bifurcation is also observed between the *M*_FC_(*T*) and *M*_ZFC_(*T*) curves over a broad temperature range. Such a bifurcation suggests a cluster glass-like behavior in the (La_0.6_Pr_0.4_)_0.67_Ca_0.33_MnO_3_ nanoparticles [38, 39]. Figure 10 shows the magnetic field dependence of the magnetizations of the (La_0.6_Pr_0.4_)_0.67_Ca_0.33_MnO_3_ samples post-annealed at 800 and 1000 °C, which are measured at different temperatures. Clearly, the *M-H* hysteresis loops demonstrate that all the samples exhibit ferromagnetic behavior at low temperatures below the *T*_*C*_ (e.g., 2 and 10 K), whereas a paramagnetic behavior is observed at 300 K. Similarly, Fig. 11 shows the temperature dependence of the magnetizations of the (La_1 − x_Pr_x_)_0.67_Ca_0.33_MnO_3_ (*x* = 0.1–0.4) nanoparticles post-annealed at 800 °C for 5 h, which were measured under ZFC mode and FC mode with an external magnetic field of 0.01 T. A PM to FM transition upon cooling was observed in the *M*_ZFC_(*T*) curves, and the *T*_*C*_ values were measured to be 233, 228, 180, and 171 K for the LPCMO samples (*x* = 0.1, 0.2, 0.3, and 0.4), respectively. Details are seen in Table 3. As compared with the La_0.67_Ca_0.33_MnO_3_ nanoparticles synthesized by sol-gel route and sintered at 800 °C in air atmosphere for 4 h (*T*_*c*_ = 253 K) [40], the *T*_*C*_ values of the (La_1 − x_Pr_x_)_0.67_Ca_0.33_MnO_3_ samples were reduced with increasing the Pr-doping concentration. The M-H loops of the (La_1 − x_Pr_x_)_0.67_Ca_0.33_MnO_3_ (x = 0.1–0.4) nanoparticles post-annealed at 800 °C for 5 h, are shown in Fig. 12. They exhibit ferromagnetic behavior at low temperatures (e.g., 2 and 10 K), but a paramagnetic behavior at 300 K. The saturation magnetization (*M*_*s*_), remanent magnetization (*M*_*R*_), and coercive field (*H*_*c*_) were obtained from the enlarged local *M-H* hysteresis loops measured at 10 K (shown in Fig. 12b, b d, f, and h, respectively), which are presented in Table 3.

**Table 3:** The Curie temperature (*T*_*c*_), saturation magnetization (*M*_*S*_), remanent magnetization (*M*_*R*_), and coercive field (*H*_*C*_) of the (La_1 − x_Pr_x_)_0.67_Ca_0.33_MnO_3_ nanoparticles post-annealed at 800 °C with different Pr-doped concentrations (*x* = 0.1–0.4)

Sample no.	Pr-doped concentration (_*X*_)	Curie temperature *T*_*C*_ (°C)	*M*_*S*_ (emu/g)	*M*_*R*_ (emu/g)	*H*_*C*_ (Oe)
B′	0.1	233 ± 1	81 ± 0.5	16.5 ± 0.1	390 ± 0.5
C′	0.2	228 ± 1	82 ± 0.5	14.2 ± 0.1	405 ± 0.5
D′	0.3	180 ± 1	61 ± 0.5	23.0 ± 0.1	585 ± 0.5
E′	0.4	171 ± 1	60 ± 0.5	15.7 ± 0.1	621 ± 0.5

Based on the above structural data obtained from XRD patterns, the unit cell volumes of the (La_1 − x_Pr_x_)_0.67_Ca_0.33_MnO_3_ (*x* = 0.1–0.4) nanoparticles are found to be decreased with increasing the Pr-doping concentration, as demonstrated in Table 2. That is ascribed to the ionic radius of Pr^3+^ (99.0 pm) being smaller than that of La^3+^ (103.2 pm) ion. As a consequence, the volume of the MnO_6_ octahedron in an orthorhombic perovskite structure is reduced with increasing the Pr-doping concentration. Thus, the Mn–O bond length in the MnO_6_ octahedron becomes shorter, which leads to the blue-shift of the stretching vibration mode frequency in the FITR spectra. From the magnetic data, it is observed that the *T*_*c*_ values of the (La_1 − x_Pr_x_)_0.67_Ca_0.33_MnO_3_ (*x* = 0.1–0.4) nanoparticles are decreased with increasing the Pr-doping concentration, which is similar to the system of the La_0.7 − x_Pr_x_Ca_0.3_MnO_3_ (*x* = 0.0–0.45) [37]. It is reported that the *e*_*g*_ electron bandwidth became narrow as the Pr-doping concentration was increased in the La_0.7 − x_Pr_x_Ca_0.3_MnO_3_ (*x* = 0.0–0.45) system, and the electron-phonon interaction was increased, resulting in a reduction of the mobility of *e*_*g*_ electrons [37]. Therefore, the double-exchange interactions in the (La_1 − x_Pr_x_)_0.67_Ca_0.33_MnO_3_ (*x* = 0.1–0.4) nanoparticles became weakened due to the narrower bandwidth and the reduced mobility of *e*_*g*_ electrons as the Pr-doping concentration was increased. This leads to a decrease of *T*_*c*_ as increasing Pr-doping concentration. It is also noticed that as the Pr-doping concentration is increased, the saturation magnetization (*M*_*s*_) is generally decreased whereas the coercive field (*H*_*c*_) is increased. Since the magnetic properties of perovskite manganites are very sensitive to the Mn–O bond length and the Mn–O–Mn bond angle, the magnetization of samples with a reduced Mn–O bond length (confirmed by a blue-shift of the stretching vibration mode frequency in the FITR spectra) is more difficult to saturate as the Pr-doping concentration is increased. This could be ascribed to the competition between the double exchange and super-exchange interactions, which leads to the canting of the manganese moments [34]. Similarly, since the ferromagnetic double exchange interaction weakens and the charge ordered clusters become more stable with increasing the Pr-doping concentration, as a result, much higher magnetic field (*H*) is required to destroy the charge ordering [38]. Thus, the increased coercive field (*H*_*c*_) is observed in the (La_1 − x_Pr_x_)_0.67_Ca_0.33_MnO_3_ (*x* = 0.1–0.4) nanoparticles as increasing the Pr-doping concentration.

## Conclusions

In summary, structural measurements based on X-ray diffraction, TEM, HRTEM, and SAED patterns provide information regarding the phase formation and the single-crystalline nature of the Pr-doping perovskite manganite (La_1 − x_Pr_x_)_0.67_Ca_0.33_MnO_3_ (LPCMO, *x* = 0.0–0.5) nanoparticles synthesized via sol-gel process. It is found that all the synthesized LPCMO samples crystallize in perovskite crystallography with an orthorhombic distortional structure, where the MnO_6_ octahedron is elongated along the *b* axis, as confirmed by Raman spectra. Lattice fringes with inter-planar spacing of 0.26 or 0.27 nm are observed in the HRTEM images taken from individual LPCMO nanoparticles, revealing the single-crystalline nature of the LPCMO nanoparticles. Fourier transform infrared spectra confirm the Mn–O bond vibrating in the stretching mode near 600 cm^− 1^ in the MnO_6_ octahedron, and this vibration mode frequency exhibits a blue shift due to the reduction of the Mn–O bong length as the post-annealing temperature or the Pr-doping concentration is increased, indicating further distortion of the MnO_6_ octahedron. XPS spectra indicate that Mn exists in a dual oxidation state (Mn^3+^ and Mn^4+^) in the LPCMO nanoparticles. Bandgaps of the LPCMO nanoparticles estimated from UV-vis absorption spectra, are in the range of 2.55–2.71 eV, indicating the semiconducting nature of the LPCMO nanoparticles. Magnetic behaviors show that all the samples undergo a PM-FM phase transition. The Curie temperatures (*T*_*c*_) of the LPCMO nanoparticles are decreased with increasing the Pr-doping concentration. The M–H hysteresis loops measured at different temperatures demonstrate that all the samples exhibit ferromagnetic behavior at 2 and 10 K, whereas paramagnetic behavior is observed at 300 K. The magnetic measurements provide a Curie temperature around 230 K, saturation magnetization (*M*_*s*_) of about 81 emu/g, and coercive field of 390 Oe at 10 K. These strong magnetic behaviors as well as their semiconducting nature will enable the LPCMO nanoparticles to be a suitable candidate used for magnetic semiconductor devices.
